# The future outlook on allergen immunotherapy in children: 2018 and beyond

**DOI:** 10.1186/s13052-018-0519-4

**Published:** 2018-07-11

**Authors:** Stefania Arasi, Giovanni Corsello, Alberto Villani, Giovanni Battista Pajno

**Affiliations:** 1 Allergy Unit- Department of Pediatrics, University of Messina, Messina, Italy; 2 SIAF- Schweizerischers Institut für Allergie-und Asthmaforschung, Davos, Switzerland; 3 Pediatric Allergy Unit, Bambino Gesù Children’s Hospital, IRCCS, Rome, Italy; 4 Department of Maternal and Child Health, University of Palermo, Palermo, Italy; 5 Pediatric and Infectious Disease Unit, Bambino Gesù Children’s Hospital, IRCCS, Rome, Italy

**Keywords:** Allergen-specific immunotherapy, Allergic rhinitis, Allergy, Children, Food allergy, IgE-mediated allergic diseases, Oral immunotherapy, Prevention, Sub-lingual immunotherapy, Sub-cutaneous immunotherapy

## Abstract

Allergen immunotherapy (AIT) is the only currently available immune-modifying and aetiological treatment for patients suffering from IgE-mediated diseases. In childhood, it represents a suitable therapeutic option to intervene during the early phases of respiratory allergic diseases such as rhino-conjunctivitis and asthma, which is when their progression may be more easily influenced. A growing body of evidence shows that oral immunotherapy represents a promising treatment option in children with persistent IgE- mediated food allergy. The efficacy of AIT is under investigation also in patients with extrinsic atopic dermatitis, currently with controversial results. Furthermore, AIT might be a strategy to prevent the development of a new sensitization or of a (new) allergic disease. However, there are still some methodological criticisms, such as: a) the regimen of administration and the amount of the maintenance dose are both largely variable; b) the protocols of administration are not standardized; c) the description and classification of side effects is variable among studies and needs to be standardized; d) quality of life and evaluation of health economics are overall missing. All these aspects make difficult to compare each study with another. In addition, the content of major allergen(s) remains largely variable among manufacturers and the availability of AIT products differences among countries. The interest and the attention to AIT treatment are currently fervent and increasing. Well-designed studies are awaited in the near future in order to overcome the current gaps in the evidence and furtherly promote implementation strategies.

## Background

**Fig. 1:**
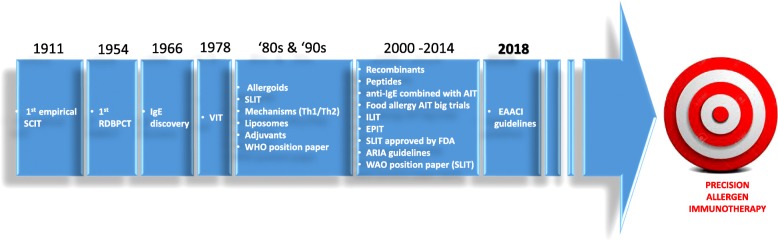
Milestones in Allergen ImmunoTherapy’s history. AIT, Allergen ImmunoTherapy; EPIT, Epicutaneous ImmunoTherapy; FDA, Food and drug administration; IgE, immunoglobulin E; ILIT, Intralymphatic ImmunoTherapy; RDBPCT, Randomized, Double-Blind, Placebo-Controlled Trial; SCIT, Subcutaneous ImmunoTherapy; SLIT, Sublingual ImmunoTherapy; Th, T cells helper; VIT, Venom Allergen ImmunoTherapy; WAO, World Allergy Organization; WHO, World Health Organization

It is estimated that more than one third of population all over the world is currently suffering from at least one allergic disease [1]. In particular, allergic rhinitis, asthma, and food allergy represent major disorders. Their incidence is increasing especially in children and young adults, who are bearing the greatest burden of these trends together with their families and health services [1]. Nowadays, most patients have good disease control and acceptable quality of life through avoidance strategies and symptomatic drug therapy. However, a minority still have persistent symptoms or remain at risk of life-threatening allergic reactions. Allergen-specific immunotherapy (AIT) is currently recognized as the only clinically effective treatment capable of a disease-modifying effect for IgE-mediated allergic diseases [1–8]. AIT may not only desensitize a patient -including who is not responsive to avoidance strategies or pharmacotherapy- thereby ameliorating symptoms while on treatment, but also deliver long-term clinical benefits that may persist for years post-AIT discontinuation. Since the first description of the clinical efficacy of subcutaneous injections of a pollen extract in hay-fever, reported by Leonard Noon in 1911 [9], AIT has been performed (Fig. 1). Typically the subcutaneous, sublingual or oral routes are used. Others, such as the epicutaneous and the intra-lymphatic ones are under investigation. In the early years, allergenic extracts of poor quality and definition were used. Substantial progress in understanding the patho-mechanisms of allergic reactions has led to improve both safety and efficacy profile of AIT in clinical practice. Currently, AIT is accepted and routinely prescribed worldwide in the pediatric population for respiratory allergies and more and more in food allergies. However, there are still several gaps to be filled, particularly around AIT long-term benefit and its use in children. The efficacy of AIT is under investigation also in patients with extrinsic atopic dermatitis, currently with controversial results [10, 11]. A better understanding in mechanisms of action of AIT might improve both the clinical efficacy of the treatment – while permitting shorter, safer and more convenient strategies for the patient- and the early or even preliminary recognition of AIT-responders. Well-designed large scale studies are still needed in order to make AIT a precision medicine, targeted to the patient.

In the text below, we preliminary synthesize the current knowledge of the mechanisms of action of AIT. Afterwards, we describe the current evidence on AIT in terms of prevention, allergic rhinitis and food allergy. Finally, the current gaps and plans to address them will be discussed.

## Mechanisms of action of AIT and predictive biomarkers

**Fig. 2:**
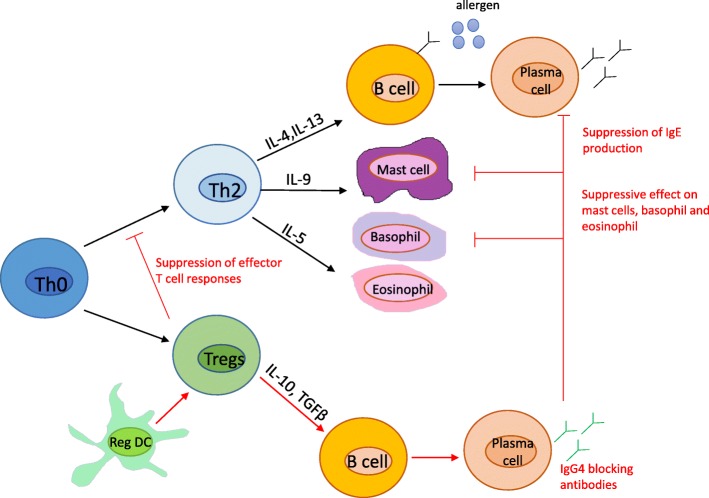
Proposed immunological mechanisms of action of immunotherapy: induction of Treg; production of IL-10 and TGF-β, cytokines to upregulate regulatory dendritic cell (regDC) and immunomodulate target cells, such as B cells, mast cells/basophils with with downregulation of IgE production by the production of IgG4, which are ‘blocking antibodies’

AIT works through several immunological pathways [12, 13]. The mechanisms of action include the induction of very early desensitization of mast cells and basophils [14, 15]; generation of specific regulatory T and regulatory B cell responses [16, 17]; regulation of allergen specific IgE, IgG4 and IgA [18–21]; decreases in numbers and activity of effector cells in mucosal of target organs, including mast cells [22], basophils [23], eosinophils [24], and type 2 innate lymphoid cells [25]; and decreases in the activity of basophils in circulation [9] (Fig. 2). However, a detailed knowledge of the mechanism involved in effective AIT is still missing. Furthermore, it is not clear whether the altered long-term memory resides within the T-cell or the B-cell compartment. Understanding mechanisms underlying induction and persistence of tolerance is a key point in order both to identify novel and more effective strategies tailored on the individual pattern and to establish predictive biomarkers of clinical response. So far, several biomarkers candidates have been investigated: IgE [total IgE, specific IgE (sIgE) and sIgE/Total IgE ratio); IgG-subclasses (sIgG1, sIgG4 including sIgE/IgG4 ratio); serum inhibitory activity for IgE (IgE-FAB and IgE-BF); basophil activation; cytokines and chemokines; cellular markers (T regulatory cells, B regulatory cells and dendritic cells) and in vivo biomarkers (e.g. provocation tests) [26]. In particolar, IgE specific activity (ratio specific IgE/total IgE) and serum IgE-FAB are currently considered as potential surrogate candidate biomarkers; however data are discordant [26]. To explore the use of allergen-specific IgG4 is recommended as a biomarker for compliance. More studies for confirmation and interpretation of the possible association with the clinical response to AIT are still needed.

## Status of the art, unmet needs and future perspectives

### General considerations

Several studies have investigated the efficacy and safety of AIT [5–7]. However, to interprete the current evidence remains challenging for the deep heterogeneity among studies. For instance, they are evaluating different populations. It is known that atopic heredity play a role in the risk of developping allergic disease(s). Furthermore, children with atopic sensitization and/or early manifestations of atopic diseases (such as atopic dermatitis and food allergy) have a higher risk for development of other allergic manifestations (e.g. asthma) [27–29]. The age of the population is also a pivotal factor as the phenotypic expression may change with age and some manifestations may even disappear spontaneously [27–29]. The results of individual studies are difficult to compare because studies have used not only different populations, but also different methods (e.g. diagnostic criteria; allergens, formulation, and strength of products used; schedules; dose; route of administration; duration of the intervention) and outcomes. Additionally, many studies have small sample size and missing adjustment for confounders. Furthermore, not all AIT products used provide sufficient data to support their efficacy in clinical practice. Therefore, an individual product-based evaluation of the evidence for efficacy is strongly recommended before treatment with a specific product is initiated [5–7]. The identification of the gaps in the current evidence is a preliminary and mandatory phase in order to stimulate in the near future the development of longitudinal, prospective, well-designed studies with the final goal of a “precision medicine/prevention”, tailored on each individual.

### Prevention

Prevention is one of the major concerns, above all in pediatrics. Furthermore, it is known that the clinical expression of respiratory allergies tends to change over time, according to a “natural history”, the so-called “atopic march”. In the typical sequence, allergic rhinitis often precedes the onset of asthma and, therefore, it can be considered a risk factor for the development of allergic asthma [27–29]. In addition, there is often the tendency to develop new sensitivities along time: the natural history of sensitizations begins usually with foods, continues with environmental allergens (usually dust mites) and ends with pollens. However, some individuals begin their march only with sensitization to mites, pollens or molds without food allergens [30]. Furthermore, molecular-based diagnostics showed that in most children the IgE response to a single allergenic source evolves over time, becoming more and more complex: the serum concentration of IgE antibodies rises progressively, both for an increase sensitizing molecules, and for a rising concentration of IgE antibodies directed against any individual allergenic molecule (the so-called phenomenon of “molecular spreading”) [30]. Interestingly, a ‘pre-clinical’ IgE sensitization has been shown already years before (up to 5 years before) the development of seasonal allergic rhinitis, initially characterized by weak and simple IgE responses, progressively increasing in concentration and molecular complexity [30, 31]. Therefore, as AIT is the only disease-modifying treatment in allergic diseases the potential preventing effects of AIT have been suggested and investigated for the prevention not only of the development of allergic comorbidities in patients with established allergic diseases, but also the development of first allergic disease in not-sensitized children (“primary immune-prophylaxis”) and in still healthy children with specific IgE antibodies (“secondary immune-prophylaxis”) and allergic sensitization in patients with other allergic conditions (“tertiary immune-prophylaxis of atopy”) [4]. Certainly, alongside efficacy, another pivotal issue to be considered is the safety profile, especially in the context of prevention in healthy individuals.

The current evidence suggests that a three-year-long course of subcutaneous or sublingual AIT can be recommended for children and adolescents with moderate to severe AR due to grass or birch pollen in order to prevent the onset of allergic asthma for up to 2 years post-AIT cessation in addition to its sustained effect on AR symptoms and medication [4, 32–37]. However, the strength of this recommendation is moderate as based on significant results from two moderate [33, 35] and two high risk of bias [32, 34] RCTs and some controlled before and after (CBA) studies. A few trials suggest a preventive effect on the onset of asthma symptoms and medication use longer than 2 years post-AIT [34, 35]. However, there is lack of evidence for AR triggered by house dust mites or other allergens different from grass/birch [4, 34, 38]. Overall, because of inconsistent results, AIT cannot currently be recommended for the prevention of new sensitizations, nor in patients with allergic rhinitis and/or asthma nor in healthy individuals [4, 34, 39–41]. For lack of evidence, no recommendation can be made in favor or against AIT in individuals with early life atopic manifestation, such as atopic eczema and food allergy nor in healthy subjects -with or without atopic sensitization- for the prevention of onset of allergic diseases [4, 42]. Therefore, though there is evidence for the preventive potential of AIT as disease modifying treatment, further well-designed clinical trials are needed to confirm the possible value of AIT in prevention of allergic diseases. They should consider the safety profile, the health-economic aspects, and the quality of life, too (Table 1). Additionally, strategies need to be targeted to different scenarios, e.g. women planning pregnancy to take preventive measures such as AIT to reduce the risk that their child will develop allergies, healthy infants and young children with atopic dermatitis and food allergy, older children with AR, healthy (with or without atopic sensitization) adolescents/adults and adolescents/adults with established allergic disease.

**Table 1:** Gaps in the evidence of AIT for prevention

Major gaps in the evidence of prevention	
❖ Long-term effectiveness of AIT in preventing asthma in children with AR due to grass pollen	
❖ Effectiveness of AIT in preventing asthma in children with AR due to house dust mites	
❖ Identification of the optimal age for introduction of AIT for prevention	
❖ Identification of the optimal duration of AIT for prevention	
❖ Identification of the optimal product, administration form, dose and schedule of AIT for prevention	
❖ Evaluation of healthy economics of AIT for prevention	
❖ Evaluation of acceptability of AIT for prevention in different patient groups (age, pattern of sensitization and clinical characteristics) and healthy individuals	
❖ Identification of the most suitable candidates	
❖ “Precision preventive medicine” algorithms	

### Allergic rhinitis

AIT is a therapeutic option in patients suffering from allergic rhinitis/rhino-conjunctivitis with/without allergic asthma with an evidence of specific IgE-sensitization towards clinically relevant inhalant allergen(s) [2, 5, 43]. It is indicated in the presence of moderate to severe symptoms interfering with usual daily activities or sleep (e.g. Allergic Rhinitis and its Impact on Asthma, ARIA) [44] despite avoidance measures and pharmacotherapy [2, 5].

Since AIT is allergen-specific, its efficacy and effectiveness depends on a proper identification of the triggering allergen(s). This concept fits into the perspective of a “precision medicine” and implies a proper recording of the clinical history and ascertainment of environmental exposure [45], confirmed by diagnostic tests [46]. Before prescribing AIT, any specific patient-related (e.g. uncontrolled or severe asthma and adherence to the treatment) and product-specific absolute or relative contraindications should be considered.

Sublingual (SLIT) and subcutaneous (SCIT) allergen immunotherapy constitutes the preferred route of administration of AIT for respiratory allergies. Alternative modalities of delivery [such as epicutaneous [47], intradermal [48] and intralymphatic routes [49]] have been recently under investigation, however with currently modest body of evidence [2]. In general, the current evidence suggests that both SCIT and SLIT are effective for AR [2, 5]. Both route of administration were associated with reductions in symptoms and with medication use. The strength of evidence is high in adult patients but moderate in pediatric patients for lack of data [2, 5]. In particular, in children suffering from moderate to severe seasonal AR, both continuous and pre- (i.e. AIT started at least 2, preferable 4 months before the pollen season) and pre−/co-seasonal AIT are currently recommended for clinical benefit during the AIT treatment [2, 5, 50–57]. Overall, there are insufficient data to determine which of SCIT and SLIT is the most effective [2, 5]. Concerning perennial AR due to house dust mites, there is evidence for efficacy of continuous AIT (both SCIT and SLIT, the latter in form of tablet but not in aqueous solution) during the AIT treatment [2, 5, 58, 59]. The evidence for clinical benefit to pediatric patient for at least 1 year after cessation of the AIT course (the so-called “long-term efficacy”) nowadays is limited to continuous grass pollen AIT (both SLIT -tablet or solution- and SCIT) performed for a minimum of 3 years in seasonal AR due to grass pollen [2, 5, 50, 60]. No study to our knowledge have investigated the long-term efficacy of AIT in perennial AR in children however there is evidence for continuous therapy with SLIT tablet in adults with AR to house dust mite [61]. In addition, evidence to support SLIT in children with asthma due to HDM is still scarce [62]. Many factors may affect the efficacy of AIT. Some factors are related to the patient, including poly-sensitization, co-existing asthma and specific issues in pre-school age. Other factors are related to the allergen(s), such as: the standardization of allergen extracts (including common allergens- whose characterization is still missing in many commercial products and/or lacking stability, e.g. molds- and “orphan allergen”, affecting a few patients); the formulation of SLIT preparation and allergen mixtures (some allergens with enzymatic activity, such as HDM, may affect the efficacy of SLIT drops). A careful evaluation of the indications to AIT and individual product-based evaluation of the evidence for efficacy is pivotal before prescribing a specific AIT product. Standardized AIT products with documented clinical evidence of efficacy should be used when available [2, 5]. Unfortunately, among the published data there is a substantial heterogeneity in terms of the study design (particularly the different outcomes used), study population and the products evaluated. This heterogeneity -as discussed above- hampers the meta-analyses and comparison among the available data [2, 5].

Many gaps are still unmet (Table 2): more prospective multi-centre controlled trials using standardized products are awaited in order to address them. New combined approaches have been suggested and experimented in order to improve adherence and quality of life with shorter courses, whilst reducing the risk of adverse reactions and improving the effectiveness [63]. For instance, adjuvants have been added to AIT extracts [e.g. TLR-4 agonists [64–67], TLR-9 agonists [68]] with promising results in adults. Anti-IgE injections have been combined with AIT schedules with safer profile and maintained effectiveness also in children. However, this approach is expensive and there is no agreement on timing and mode of anti-IgE discontinuation when AIT maintenance is achieved [69, 70]. Another attractive approaches lies on the use of recombinant AIT as it allows accurate standardization of allergen products, and potentially a personalized treatment based on the individual allergic sensitization(s) [71]. Further studies are awaited to further investigate these interesting approaches.

**Table 2:** Gaps in the evidence of AIT for allergic rhinitis

Major gaps in the evidence of AIT for allergic rhinitis	
❖ Lack of agreement on clinically relevant outcomes of effectiveness and clinically meaningful effect size of AIT (active vs placebo)	
❖ Lack of evidence of clinical effectiveness for some products	
❖ Lack of standardized AIT preparations for “orphan allergens”	
❖ Lack of evidence for effectiveness of mixtures of homologous allergens	
❖ Evidence for long-term clinical effectiveness after discontinuation treatment	
❖ Standardization of grading of adverse effects of AIT	
❖ Approaches to minimize adverse effects	
❖ Good evidence base for contraindicating AIT	
❖ Approaches to improve adherence to AIT	
❖ Role of adjunctive treatment(s) (e.g. omalizumab)	
❖ Cost-effectiveness and cost-utility studies	
❖ Good understanding of mechanisms of action	
❖ Identification of biomarkers of response, to predict and quantify the effectiveness of AIT	
❖ Identification of the most suitable candidates	
❖ “Precision medicine” algorithms	

### Food allergy

IgE-mediated food allergy (FA) is a potentially life-threatening condition [72], with a negative impact on the quality of life of patients and their family [73, 74]. The current standard approach consists of the strict avoidance of the culprit food and rescue medication in the event of an allergic reaction occurs [75]. However, an elimination diet may be difficult and frustrating in patients with persistent FA, above all for those foods (e.g. cow’s milk, CM, and hen’s egg, HE) that are central in the common diet [75]. Nevertheless, despite efforts to comply with this diet, accidental exposures leading to adverse reactions are frequent [76, 77]. In this context, considering the potential desensitizing effects of allergen administration, AIT has been investigated. The most frequent route of administration consists of the immediate swallowing of the allergen (oral immunotherapy, OIT). On the basis of the current body of evidence, OIT is performed more and more in clinical practice, though still in a few rate of eligible patients. OIT involves the administration of increasing doses of the culprit allergen until the food is tolerated at usually dietary doses. This approach can confer protection against accidental allergic reactions and contribute to improve nutritional status and quality of life of the affected patients [74]. Many clinical trials performed with cow’s milk, hen’s egg and peanuts consistently show that an effective increase of the threshold of reaction while on OIT (desensitization) can be obtained, and therefore recommended, in children with persistent FAs, from around 4–5 years of age as most patients overcome their FAs to CM and HE spontaneously. However, it is not clearly defined if when desensitization has been achieved, a permanent tolerance persists, independent of the regular assumption of the responsible food [3, 6, 78, 79]. Adverse events may occur but most of them are not severe [6]. It can be performed only in highly specialized centers and under strict medical supervision after the informed consent has been obtained from parents [3, 6, 80–82]. Other routes of administration have been investigated (e.g. sublingual, subcutaneous and epicutaneous ones) [83–86] as well as adjunctive treatments (such as omalizumab and probiotics) [87–89]. Though AIT represents an emerging reality as an active treatment for IgE-mediated food allergies, many issues remain unanswered. Clinical trials for OIT so far conducted are extremely heterogeneous and therefore their results are not comparable. Differences encompass dosage, amount and frequency, duration of build-up and maintenance phases, type of allergen used, patient characteristics, reporting in adverse events and adjuvant therapies (Table 3) [78]. Much larger, longitudinal and well-designed studies using more homogenous protocols are needed in order to standardize products and to validate protocols (optimal doses and schedule), to assess the sustainability of the desensitization process, to improve the effectiveness after AIT discontinuation, the safety, and the impact on quality of life, and to identify the role of adjunctive therapies (such as omalizumab and probiotics) [78].

**Table 3:** Gaps in the evidence of FA-AIT

Gaps in the evidence of FA-AIT	
❖ Lack of standardized products and vehicles	
❖ Lack of validated and shared protocols	
❖ Lack of agreement on clinically relevant outcomes of effectiveness	
❖ Evidence for long-term clinical effectiveness after discontinuation treatment	
❖ Standardization of grading of adverse effects of AIT	
❖ Approaches to minimize adverse effects	
❖ Adjunctive treatment(s)	
❖ Impact on quality of life	
❖ Cost-effectiveness and cost-utility studies	
❖ Good understanding of mechanisms of action	
❖ Identification of biomarkers of response	
❖ Identification of the most suitable candidates	
❖ “Precision medicine” algorithms	

## Conclusions

Through an overview of the up-to-date evidence in terms of mechanisms of action, efficacy and safety of AIT for prevention, allergic rhinitis and food allergy, this rostrum sought to gauge the main needs currently unmet in AIT in order to stimulate in the near future the development of longitudinal, prospective, well-designed studies with the final goal of a “precision medicine” tailored on each single eligible subject [90, 91]. A deep understanding of mechanisms of action will improve the current strategies and provide new ones for immune intervention, which will likely include targeting of the molecular mechanisms of allergen tolerance and reciprocal regulation of effector and regulatory T cell subsets. The molecular-based diagnostics would certainly improve the accuracy in AIT prescription, allowing to dissect the genuine sensitizations and the cross-reactions due to pan-allergens [92]. Mobile health technologies might establish a cause-effect relationship between exposure to the pollen recognized by the patient’s IgE sensitization pattern and the patient’s symptoms and precisely assess the degree of severity of the patient’s symptoms, as AIT should be administered primarily to patients with moderate-severe rhinitis [2]. An integrated approach combining different available diagnostic tools might achieve a more precise etiological diagnosis for a better AIT prescription. However, to our best knowledge, no informatics tool dedicated to support the implementation of internationally validated algorithm is so far available. Furthermore, the development of integrated care pathways incorporating (educating and training) primary and secondary care, as well as the availability of high quality AIT products, individual product-based evaluation of the evidence, and global actions aimed to develop a harmonized international approach to regulate AIT products are awaited in order to implement AIT in clinical practice. The interest and the attention to AIT treatment are currently fervent and increasing. Well-designed studies are awaited in the near future in order to overcome the current gaps in the evidence and furtherly promote implementation strategies.

## Abbreviations

AIT: Allergen ImmunoTherapy
CBA: Controlled before and after study
CM: Cow’s Milk
FA: Food Allergy
HDM: House Dust Mite
HE: Hen’s Egg
OIT: Oral ImmunoTherapy
RCT: Randomized controlled trial
SCIT: Subcutaneous ImmunoTherapy
SLIT: Sublingual ImmunoTherapy
